# Surflex-QMOD: physically meaningful QSAR

**DOI:** 10.1186/1758-2946-6-S1-P55

**Published:** 2014-03-11

**Authors:** Alexander Steudle, Rocco Varela, Ajay N Jain

**Affiliations:** 1 Certara International, Martin-Kollar-Straße 17, 81829 München, Germany; 2 Department of Bioengineering and Therapeutic Sciences, University of California, San Francisco, California 94143-0912, USA

## 

Computational methods for predicting ligand affinity where no protein structure is known generally take the form of regression analysis based on molecular features that have only a tangential relationship to a protein/ligand binding event. Such methods have utility in retrospective rationalization of activity patterns of substituents on a common scaffold, but are limited when either multiple scaffolds are present or when ligand alignment varies significantly based on structural changes. In addition, such methods generally assume independence and additivity of effect from scaffold substituents. Collectively, these non-physical modeling assumptions sharply limit the utility of widely used QSAR approaches for prospective prediction of ligand activity.

The approach we report builds upon the Compass approach by constructing physical models of a protein binding site based upon ligand binding data. The result is a binding site composed of molecular fragments that can be treated as a target for molecular docking. The binding site model consists of molecular fragments that can account for multiple positions of protein residues. It is not a literal reconstruction of a single configuration of protein residues. New molecules are docked directly into the binding site, with their highest scoring poses serving as the prediction of binding geometry and the corresponding score being the predicted affinity. By deriving a virtual binding pocket at the same time as the relative poses of ligands are identified, the key analogy is that one can treat a computational model of a binding site as one treats a protein binding pocket. We seek the optimal fit of ligands into the binding site. One begins with a guess as to the initial alignment of ligands, then constructs a model of activity that depends on the ligands’ poses. The model can be thought of as a virtual receptor. Next, one seeks poses for each ligand that optimize their interaction with the virtual receptor. Then, the virtual receptor is refined, making use of the new ligand poses, and the process iterates between pose refinement and virtual receptor refinement. As the virtual receptor evolves, the changes in ligand scores due to pose optimization decrease. When the iterative process converges, the final poses of the ligands are optimal with respect to the final virtual receptor. The software implementing the algorithms for pocket construction and ligand activity prediction constitute a new module within the Surflex platform, called Surflex-QMOD (**Q**uantitative **Mod**eling).

The Surflex-QMOD approach addresses the physical linkage between activity model and molecular binding mode with pockets having detailed structure comparable to true protein binding sites. Because the model building process results in a model that selects ligand alignments based on mutual interaction, there is a direct correspondence between the physical process of protein/ligand binding and the act of prediction.

